# Full-Scale Measurements of Translational and Torsional Dynamics Characteristics of a High-Rise Building during Typhoon Sarika

**DOI:** 10.3390/ma15020493

**Published:** 2022-01-10

**Authors:** Jiaxing Hu, Zhengnong Li, Zhefei Zhao

**Affiliations:** 1 School of Civil and Environmental Engineering, Hunan University of Science and Engineering, Yongzhou 425199, China; 2 Key Laboratory of Building Safety and Energy Efficiency of the Ministry of Education, Hunan University, Changsha 410082, China; zhn88@263.net; 3 School of Vocational Engineering, Health and Sciences, RMIT University, GPO Box 2476, Melbourne, VIC 3001, Australia; zhefeizhao@gmail.com

**Keywords:** full-scale measurement, typhoon, high-rise building, torsional wind-induced responses, modal parameter

## Abstract

The field measurement of wind-induced response is of great significance to the wind resistance design of high-rise buildings, in particular torsional responses measured from high-rise buildings under typhoons. The measured high-rise building, with a height of 108 m, has 32 stories and is supported by giant trusses with four massive columns. Acceleration responses along translational and torsional directions were monitored synchronously and continuously during the passage of Typhoon Sarika on 18 October 2016. The wind speed and wind direction at the height of 115 m, the translational accelerations on a total of six floors and the angular accelerations on a total of four floors were recorded. The time and frequency domain characteristics of translational acceleration and torsional angular accelerations were analyzed. The amplitude-dependent translational and torsional modal frequencies of the measured building were identified by NExT-ERA, SSI, and RDT methods. The full-scale study is expected to provide useful information on the wind-resistant design of high-rise buildings in typhoon-prone regions.

## 1. Introduction

A typhoon, also known as a destructive tropical cyclone, is an intense meso-scale vortex characterized by a calm eye and a ring of violent winds in the eyewall. The southeast coast of China is a typhoon-prone area from May to November every year. Located at the southernmost part of China facing the South Sea and the Pacific Ocean to the south and east, Hainan Island is vulnerable to typhoon attacks. It is estimated that 92 tropical cyclones landed in Hainan from 1964 to 2018, with an average of nearly 2 typhoons landings each year. Thus, it is crucial to strengthen the monitoring and prediction of typhoon. Massive high-rise buildings have emerged due to the development of new materials, construction technology and analytical technology, and the dynamic response will be amplified when the height of high-rise buildings increases under the excitation of strong wind, especially under the actions of typhoons or hurricanes. Moreover, with the development of high strength materials, modern tall buildings are more flexible than those constructed with traditional materials. Therefore, the modal parameters are vital to evaluate the reliability, safety and applicability of high-rise buildings, and can be efficiently used for structural damage detection and identification.

First, field measurements on the translational dynamic response (acceleration, velocity and displacement) of high-rise buildings have widely conducted during typhoons. For example, Z N Li et al. (2018) [1] carried out measurements of wind-induced response on a 108 m building during Typhoon Kalmaegi and identified amplitude-dependent modal parameters by the ERA-NExT, RDT and Half-power bandwidth methods, the results revealed that the first three modal frequencies decreased when the acceleration amplitude increased. Li M et al. (2020) [2], Li Q S et al. (Li et al., 2017 [3], 2011 [4]) and An Xu et al. (2014) [5] conducted a series of full-scale measurements of wind-field characteristic, dynamic response on super-tall buildings (Di-Wang Tower, Jin Mao Building, Canton Tower, Guangzhou West Tower, etc.) during typhoons, which provided critical scientific data for understanding the structural dynamic characteristics (mode shapes, natural frequencies, and damping ratios) of high-rise buildings. Generally, the three-dimensional accelerometer is often used in modal parameter identifications of high-rise buildings, and the translational acceleration usually covers the east-and-west, south-and-north, and vertical vibrations. The complete motion characteristics of a rigid body at a certain point also include rotations, which includes six parts, namely three translational and three rotational degrees of freedom to describe the vibration response. Numerous findings show that the torsional parts contribute significantly to the wind-induced response, and the torsional component is certainly generated significant shear and bending moments in structural members and further cause torsional damage to building structures, especially to the slender super-tall buildings under the action of typhoons. Most of studies for wind-induced responses of high-rise buildings during typhoons emphasize the translational responses, only a few of them concern torsional responses due to the lack of torsional accelerometer equipment with the angular vibration acceleration sensors that are used for the measurement of the 6-DOF systems, especially for the prototype measurement of torsional modal parameters of high-rise buildings under the influences of typhoons have been considered. 

Second, the modal frequency have recently been hot research topics. The measured wind-induced response of high-rise buildings under typhoons usually includes displacement, velocity and acceleration, and recording acceleration signals occupy dominant in field measurement under typhoon effects. Many scholars (Tamura Y, et al., 1996 [6]; Jeary, et al., 1992 [7]; Jun Y et al., 2015 [8]; Li et al., 2007 [9], 2003 [10]) show that the modal parameters are closely related to vibration amplitude, and the vibration amplitude is not considered in the codes and standards of the United States [11], Europe [12], Japan [13], and China [14]. These will cause significant errors in estimating the translational and torsional fundamental frequency of super-tall buildings under typhoons. Thus, numerous field measurements are urgently needed to analyze the translational and torsional dynamic characteristics during the passage of typhoons, and the findings of the amplitude-dependent modal frequency are essential to the health monitoring of high-rise building.

In summary, field measurements on the torsional wind-induced responses of high-rise buildings under typhoons are necessary to evaluate wind effect of high-rise buildings, while few field measurements have been conducted on torsional modal parameters of high-rise buildings during typhoons. Therefore, the analysis of the torsional wind-induced responses of high-rise buildings has great practical significance. The measured torsional responses can accumulate valuable data for the torsional wind-resistant design of high-rise buildings under the effect of typhoon and lay a foundation for the subsequent torsional wind-resistant measurements.

## 2. Full-Scale Measurement Program

### 2.1. Typhoon Sarika

**Figure 1:**
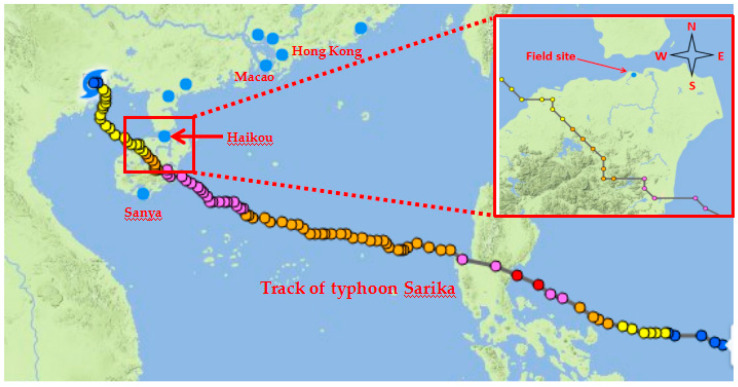
Track of typhoon Sarika and surrounding environment of field site.

As reported by Wenzhou typhoon network (http://www.wztf121.com/, accessed on 1 November 2021), Typhoon Sarika formed as a tropical depression over the western North Pacific Ocean at the East of Philippines on 12 October 2016 and moved west-northwestwards and upgraded into a severe tropical storm, then Typhoon Sarika landed on the eastern coast of Luzon Island, Philippines at 2:20 15 October 2016, reaching its peak intensity with estimated maximum sustained winds of 195 km/h and the minimum air pressure was 930 hPa. After moving westward rapidly for two days, Sarika weakened to a severe tropical storm as it made landfall along the coast of Hele Town of Wanning City, Hainan Province at 9:50 on 18 October 2016. On 19 October, Sarika was rapidly deteriorating as made its final landfall over in the coastline and border of Vietnam and China at 17:00. Typhoon Sarika affected 2.99 million people. The track of typhoon Sarika and surrounding environment of field site is illustrated in Figure 1.

**Figure 2:**
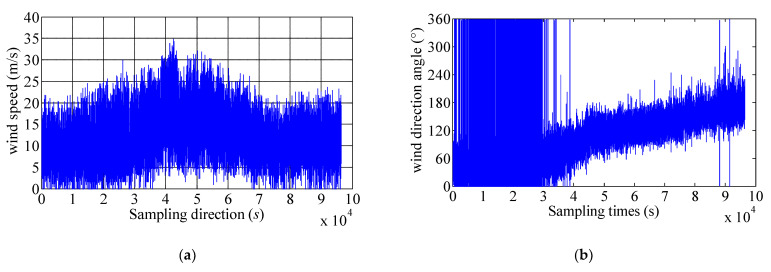
The measured instantaneous wind speed and direction during Typhoon Sarika. (**a**) Wind speed; (**b**) Wind direction.

The 36.5 h wind-field data collected beginning at 23:00 on 17 October 2016 are used to record the wind speed and wind direction during Sarika. The measured maximum instantaneous wind speed is 34.84 m/s. Before Typhoon Sarika landing, the wind direction varies greatly and shows unstable, and during the passage of Typhoon Sarika landing, wind direction changes by approximately 180°, resulting the wind direction changes from approximately northerly to southerly. During the strongest landing passage of Typhoon Sarika, the wind direction is varied within 70~110°. Figure 2 shows the measured instantaneous wind speed and wind direction during Typhoon Sarika, the peak of 10-min mean wind speed is about 20.02 m/s and the 10-min mean wind direction is within 0–180°.

### 2.2. Description of the Measured Building

**Figure 3:**
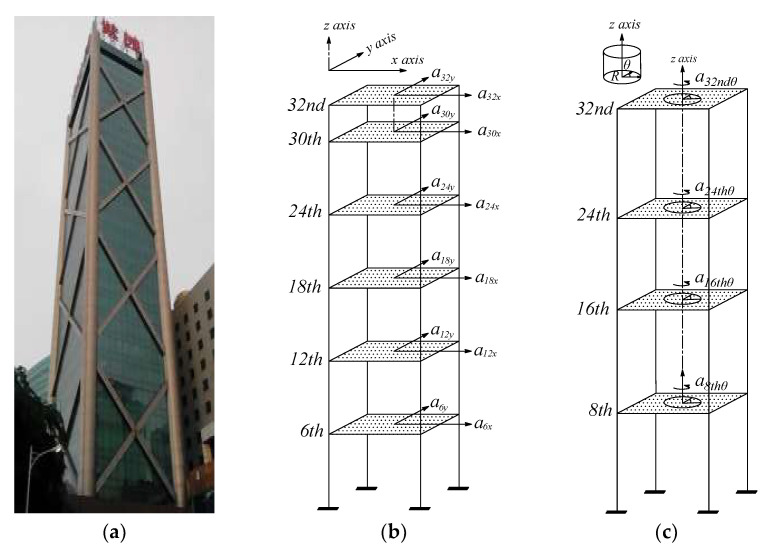
Overview of the measured building, translational and torsional measured points. (**a**) The measured building; (**b**) Translational measured points; (**c**) Torsional measured points.

The measured building lies in the typhoon-prone region with a latitude of 20°02′ N and a longitude of 110°35′ E. The measured building has a height of 108 m with the length-width ratio of 1.47, the aspect ratio between the building’s height and transverse width is about 6.71, as shown in Figure 3a.

### 2.3. Introduction of Measuring Instruments and Measuring Point Scheme

**Figure 4:**
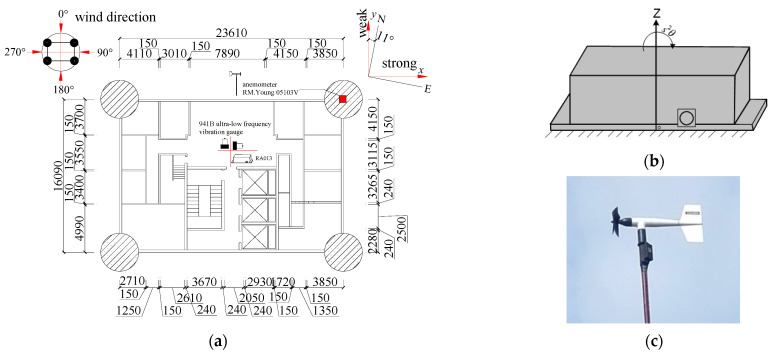
Plan view of the measured building and monitoring instruments. (**a**) Building plan; (**b**) RA013 rotational accelerometer; (**c**) Anemometer.

The time-histories of wind speed, wind direction and dynamic responses are all collected with a sampling frequency of 128 Hz by the monitoring system (Model uT33) produced by Wuhan uTekL Electronic Technology Co., Ltd. (Wuhan, China) Wind-field at a height of 115 m is measured by the mechanical anemometer (Model 05103V, See Figure 4c for details) The 0 direction of the anemometer is parallel to the *y*-axis and refers to North by West 11°, and the 90° of the anemometer represents the measured wind direction parallel to the *x*-axis, and the designation of other angles follow the clockwise rule. The translational acceleration responses of the 6th, 12th, 18th, 24th, 30th, and 32nd floors are recorded by model 941B ultra-low frequency vibration gauge produced by the Institute of Engineering Mechanics of China Earthquake Bureau with the measurement range of 0~±20 m/s^2^. The torsional angular acceleration responses of the 8th, 16th, 24th, and 32nd floors are monitored by model RA013 rotational accelerometer produced by the Institute of Engineering Mechanics of China Earthquake Administration with the measurement range of 0~±5 rad/s^2^. Theoretical analysis and experimental results show that the RA013 rotational accelerometer has good amplitude-frequency characteristics, linearity and good resistance of effects of other translational and rotational components, and the rotational accelerometer has the ability to resist transverse rotations and translations (horizontal and vertical vibrations). The installation method of RA013 rotational accelerometer, as shown in Figure 4b, measures the rotational acceleration around the Z axis. Figure 4a presents that overview of the measured building, translational and torsional measured points. Table 1 shows that specifications of the anemometer and acceleration sensors.

## 3. Dynamic Acceleration Response of the Measured Building

### 3.1. Translational Acceleration Response

**Figure 5:**
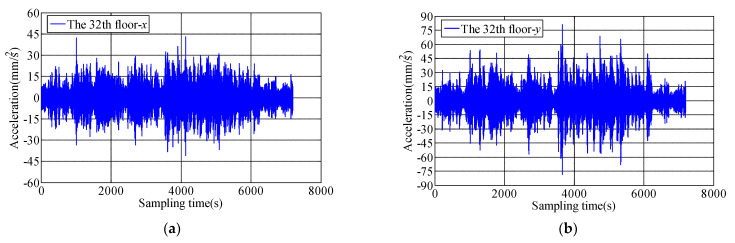
Time history of acceleration response on 32th floor during Typhoon Sarika. (**a**) 32nd floor along *x* axis; (**b**) 32nd floor along *y* axis.

**Figure 6:**
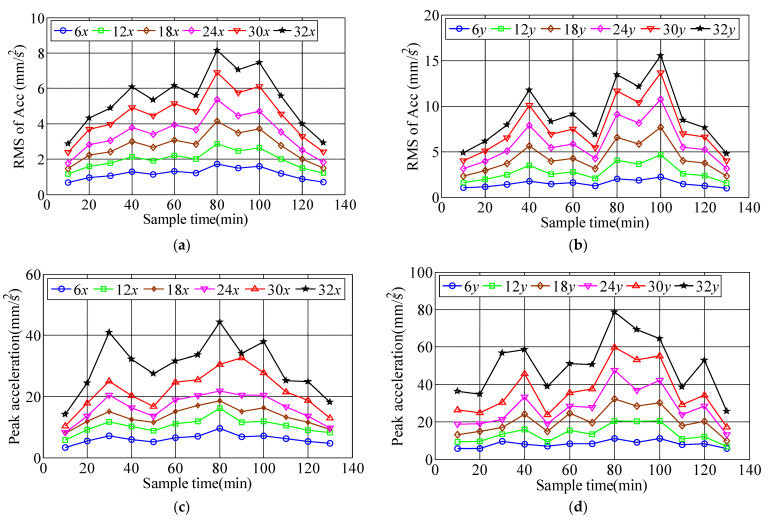
The 10-min variations of the RMS of Acc and peak accelerations along longitudinal and lateral direction during Typhoon Sarika. (**a**) RMS of Acc along *x*-axis; (**b**) RMS of Acc along *y*-axis; (**c**) Peak acceleration along *x*-axis; (**d**) Peak acceleration along *y*-axis.

The translational acceleration time-history on 6th, 12th, 18th, 24th, 30th, 32nd floors recorded by 991B translational accelerometer during Typhoon Sarika. The acceleration time histories of the top 32nd floor during typhoon landing is shown in Figure 5, the results shows that the longitudinal and lateral acceleration response increases with the increase of floor height, and the measured peak accelerations of top floor along longitudinal and lateral direction are 0.044 m/s^2^ and 0.079 m/s^2^, respectively. Vibration response of weak axis in lateral direction of measured building is greater at high wind speed than strong axis in longitudinal direction. When the peak acceleration response reaches the range of 0.050~0.150 m/s^2^, the resident will feel disturbed during the passage of Typhoon Sarika. In this study, 10-min variations of the mean-square-root of acceleration (RMS of Acc) and peak accelerations on 6th, 12th, 18th, 24th, 30th, 32nd floors are analyzed from recorded data, which show that the RMS of Acc and peak accelerations increase with the increase of floor height, Figure 6 shows the 10-min variations of the RMS of Acc and peak accelerations along both *x* and *y* axes during Typhoon Sarika.

**Figure 7:**
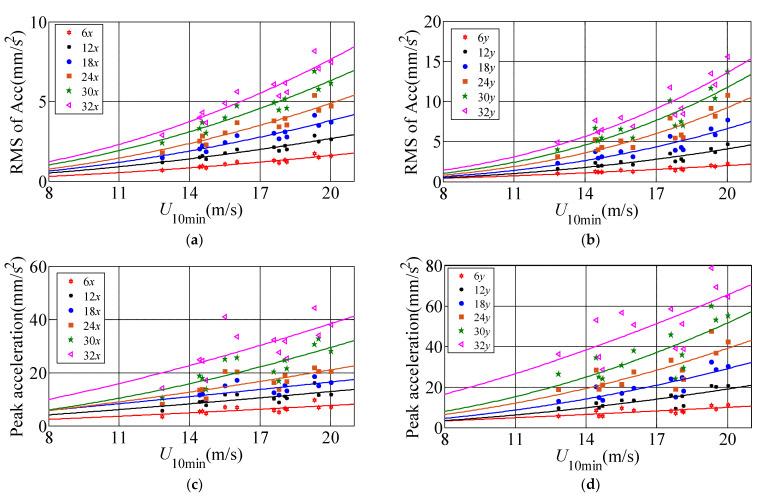
The relationship between the RMS of Acc and peak acceleration along longitudinal and lateral directions of the measured six floors with respect to the mean wind speed. (**a**) RMS of Acc along *x*-axis; (**b**) RMS of Acc along *y*-axis; (**c**) Peak acceleration along *x*-axis; (**d**) Peak acceleration along *y*-axis.

Since the wind direction of Typhoon Sarika that is close to the *x*-axis (90°) with the range of (75° 105°) during Sarika at high wind speed, therefore, the dynamic response is basically parallel to the *x*-axis in longitudinal direction and is basically parallel to the *y*-axis in lateral response. The power-law function $\sigma_{a}=c_{1}U^{c_{2}}$ is applied to the 10-min RMS of Acc and peak accelerations related to the mean wind speed. Where $\sigma_{a}$ is the RMS of Acc or peak acceleration, c_1_ and c_2_ are constants, which are determined by the measured acceleration samples under different wind speeds, and the constants of c_1_ and c_2_ on 6th, 12th, 18th, 24th, 30th, 32nd floors are shown in Table 2, and the fitting unit is mm/s^2^. Figure 7 shows the relationship between the RMS of Acc and peak acceleration along longitudinal and lateral directions of the measured six floors with respect to the mean wind speed under the action of typhoon Sarika. The results show that the RMS of Acc and peak accelerations along longitudinal and lateral directions increases a power-law function with the increase of the mean wind speed. Namely, the longitudinal (along *x*-axis) and lateral (along *y*-axis) acceleration response during Typhoon Sarika are consist well with a power-law function with respect to the increases of wind speed.

### 3.2. Torsional Angular Acceleration

**Figure 8:**
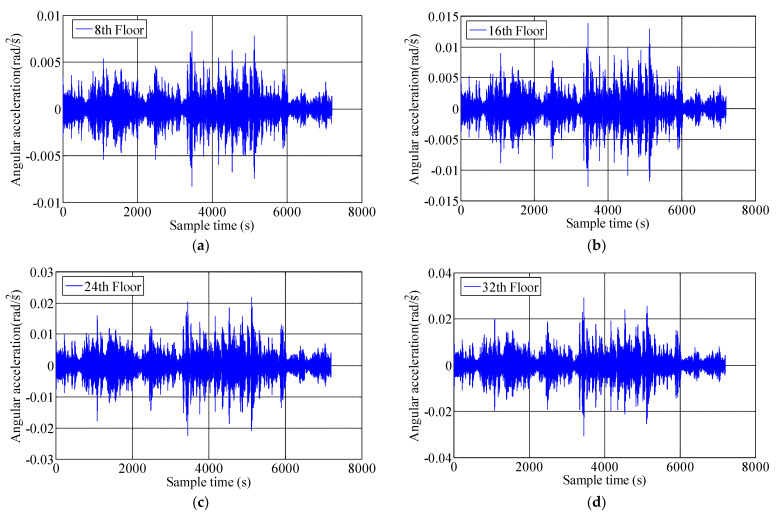
The time-history response of torsional angular accelerations on 8th, 16th, 24th and 32nd floors during Typhoon Sarika. (**a**) 8th floor; (**b**) 16th floor; (**c**) 24th floor; (**d**) 32nd floor.

**Figure 9:**
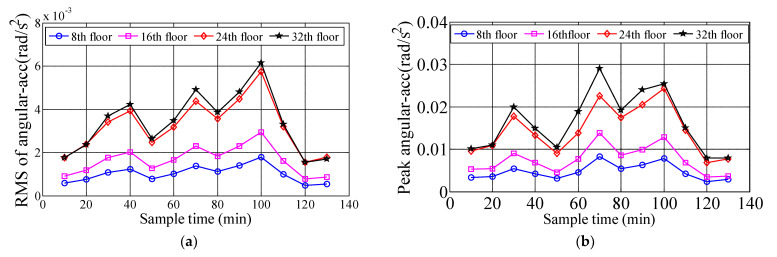
The 10-min variation of RMS of angular-Acc and peak angular accelerations on different floors. (**a**) RMS of angular-Acc; (**b**) Peak angular accelerations.

**Figure 10:**
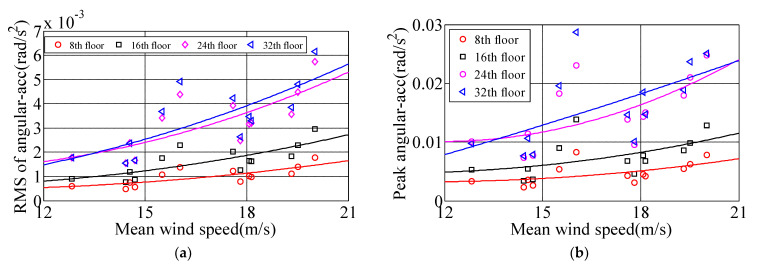
The relationship between the RMS of angular-Acc and peak angular acceleration with respect to the mean wind speed. (**a**) RMS of angular-Acc; (**b**) Peak angular accelerations.

In order to obtain the torsional angular acceleration response of measured high-rise buildings under the influence of typhoon Sarika, the RA013 torsional accelerometer was installed near the center of 8th, 16th, 24th and 32nd floors, which recorded the time-history response of the torsional angular acceleration of four floors under Sarika. Figure 8 shows the time-history response of torsional angular acceleration on 8th, 16th, 24th and 32nd floors during typhoon attack, the measured maximum torsional angular acceleration is 0.031 rad/s^2^ of the top floor, and Figure 9 show that the 10-min variation of RMS of angular accelerations (RMS of angular-Acc) and peak angular accelerations on 8th, 16th, 24th and 32nd floors, the results show that the RMS of angular-Acc and peak angular acceleration of high-rise buildings under typhoon action are very significant and the torsional response is a governing factor in designing high-rise building. A quadratic function $\sigma_{a}=c_{1}U^{2}+c_{2}U+c_{3}$ is deployed to fiting the relationship of RMS of angular-Acc and peak angular acceleration with respect to the 10-min mean wind speed. Where $\sigma_{a}$ is the RMS of angular-Acc or peak angular acceleration, c_1,_ c_2_ and c_3_ are constants, which are determined by the measured acceleration samples under different wind speeds and the constants of c_1,_ c_2_ and c_3_ on 8th, 16th, 24th, 32nd floors are shown in Table 3, and the fitting unit of angular acceleration is rad/s^2^. Figure 10 shows the relationship between the RMS of angular-Acc or peak angular acceleration of the measured four floors with respect to the 10-min mean wind speed during typhoon Sarika. The results show that the RMS of angular-Acc and peak angular acceleration increases a quadratic function as the wind speed increases, which is similar to variation of the translational acceleration response during typhoon effect.

### 3.3. Frequency-Domain Characteristics of Translational and Torsional Dynamic Response

**Figure 11:**
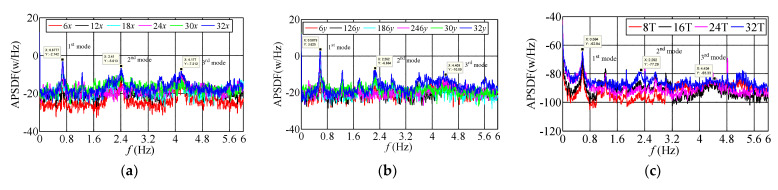
The measured APSDF when *U*_10min_ = 2.60 m/s. (**a**) APSDF along *x*-axis; (**b**) APSDF along *y*-axis; (**c**) APSDF along torsional direction.

**Figure 12:**
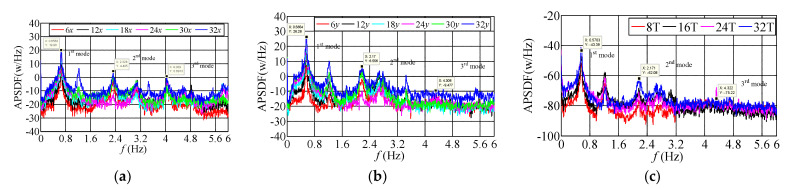
The measured APSDF when *U*_10min_ = 12.83 m/s. (**a**) APSDF along *x*-axis; (**b**) APSDF along *y*-axis; (**c**) APSDF along torsional direction.

**Figure 13:**
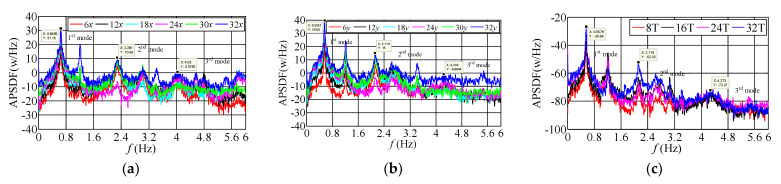
The measured APSDF when *U*_10min_ = 20.02 m/s. (**a**) APSDF along *x*-axis; (**b**) APSDF along *y*-axis; (**c**) APSDF along torsional direction.

The auto-power spectral density function (APSDF) is a critical parameter of the random vibration frequency characteristics; it can show the frequency-domain characteristics of the measured building. In addition, the average periodogram method indicates the average characteristics of statistical parameters, which is a method to analyze the frequency domain characteristic of the object building. The recorded sample duration overlapped with each other is firstly divided into several segments, and 50% of the overlapped segments are selected and processed by the window function. The APSDF of the signal can be obtained by the average periodogram method:

(1) $$S_{xx}(k)=\frac{1}{MN_{FFT}}\sum_{i=1}^{M}X_{i}(k)X_{i}^{*}(k)$$

where $X_{i}(k)$ is the Fourier transform of the one-dimensional random vibration signal of a measuring point at the *i*th data segment; $X_{i}^{*}(k)$ is the conjugate complex of $X_{i}(k)$; $N_{FFT}$ is the data length of Fourier transform; *M* is the average time to compare the frequency-domain characteristics of the measured acceleration signals at different stages of typhoon landing, three 10-min translational and torsional acceleration samples with slight difference of wind direction are selected when the mean wind speeds are 2.60 m/s, 12.83 m/s, 20.02 m/s in sequence, and the absolute value of wind direction angle along *x*-axis is less than 20°, which can be approximated that the dynamic response along *x* and *y* axes is mainly affected by along-wind and cross-wind loads, respectively. To show the measured APSDF of translational and torsional acceleration on different floors well, the logarithmic spectrum used in ordinates, and the identification results of APSDF by the average periodogram method shown in Figure 11, Figure 12 and Figure 13. The power spectral density functions of both translational acceleration and torsional angular acceleration appeared the peak values when the frequency approximates to 0, which are mainly caused by the interference of the measuring equipment and low-frequency signals. However, the phenomenon does not affect the identification and analysis of the first three modal parameters. The results show that: ① under the same wind speed excitation, the peak value of the self-power spectrum corresponding to the modal frequency along *y*-axis (weak-axis along cross-wind direction) is obviously more extensive than that along *x*-axis (strong-axis along-wind direction). Self-power spectrum, which coincides with the recorded acceleration amplitude along *y*-axis is greater than that along *x*-axis, indicating that the cross-wind dynamic response under typhoon action is greater than the along-wind dynamic response under certain conditions. ② Torsional dynamic response cannot be neglected, and the angular acceleration under typhoon action is obviously more substantial than that under static wind action. The peak values of self-power spectrum along torsional direction (T) corresponding to the first three order mode frequencies are less than those along the translational direction, which indicates that the torsional vibration energy under typhoon action is less than that along translational direction. ③ Since the energy distribution of fluctuating wind concentrated in the low-frequency region, it has a significant influence on the measured low-order modal frequencies of high-rise buildings. The measured results present that the peak values of APSDF corresponding to the first three modal frequency, especially those at the first modal frequency along translational and torsional directions increase significantly with the increasing wind speed during typhoon landing, which indicates that the first order vibration mode is the dominant mode along *x* and *y* axes and torsional direction under typhoon action. ④ The frequencies corresponding to the peak values of APSDF under the ambient excitation are more prominent than that under the action of strong wind, which explains that the translational and torsional amplitude-dependent modal frequencies of the first three orders decrease significantly as the wind speed increases.

## 4. Modal Parameter Identifications

According to the Eigen-system Realization Algorithm (ERA) and Natural Excitation Technique (NExT), ERA-NExT is often adopted to widely used in modal parameter identifications under typhoons (Chang et al., 1974 [15]; Chang and Pakzad, 2013 [16]). NExT was proposed by James et al. (1993 [17]) to identify parameters. The method is widely applied to modal testing on structural responses according to the cross-correlation function. The original ERA method (Juang and Pappa, 1985) [18] adopts a system’s impulse response (i.e., Markov parameter) to improve the controllability and observability. Based on the inputs of several cross-correlation functions, the ERA-NExT is adopted to form parameter models and identify modal parameter as well. Stochastic subspace identification (SSI) (Bart P, 2000 [19]; Bart P and Roeck D, 1999 [20]; 2001 [21]) is an advanced identification method at present, the method is according to the continuous-time deterministic state-space model, and assumption that the system is excited by white noise, the matrix QR-factorization and SVD are applied to identify the discrete-time state vector of the system, then the modal parameters of the structure are obtained by calculating the eigenvalues and eigenvectors of the matrix.

In the process of time-domain algorithm identification, the numbers of system orders are always a difficult problem to choose. Stability diagram method is currently employed to determine the orders of the system. The basic theory is that assuming that the model has different orders, the state space models of different orders can be obtained. The modal parameters of each model orders can be identified. All the calculated system orders can be drawn on a diagram to help identify the modal parameters. The modal parameters of different system orders are plotted in the same diagram, so on the axis corresponding to a certain mode, the identification parameters of the higher model orders are compared with those of the lower model orders, if the difference of modal frequency, damping ratio and mode shape of the system is less than a given value in advance. Then this point is the stabilization point. The axis composed of these points is the stabilization axis, and the corresponding modes can be judged as the modes of the system. At present, the stability diagram method is generally considered to be a useful method to determine the order of the system.

**Figure 14:**
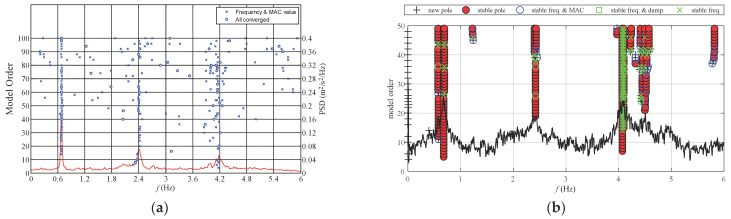
The stability diagram identified by ERA-NExT and SSI. (**a**) ERA-NExT along *x*-axis; (**b**) SSI along *y*-axis.

In this paper, the power spectral density (PSD) and stability diagram are combined to determine the order of the system. When the frequency difference between two adjacent model orders is within a certain range, and the eigenvalue is stable, and the PSD with respect to this eigenvalue has obvious peaks, the real mode can be distinguished from the noise mode by combining the PSD diagram with the stability diagram. The NExT-ERA and SSI methods are used to identify the stability diagram under static wind condition, and the PSD diagram was drawn together with the stability diagram. Figure 14 shows that the more points appear near the eigenvalue, the more real the eigenvalue is, and the combination of PSD and the stability diagram can effectively identify the stable points of the eigenvalue.

**Figure 15:**
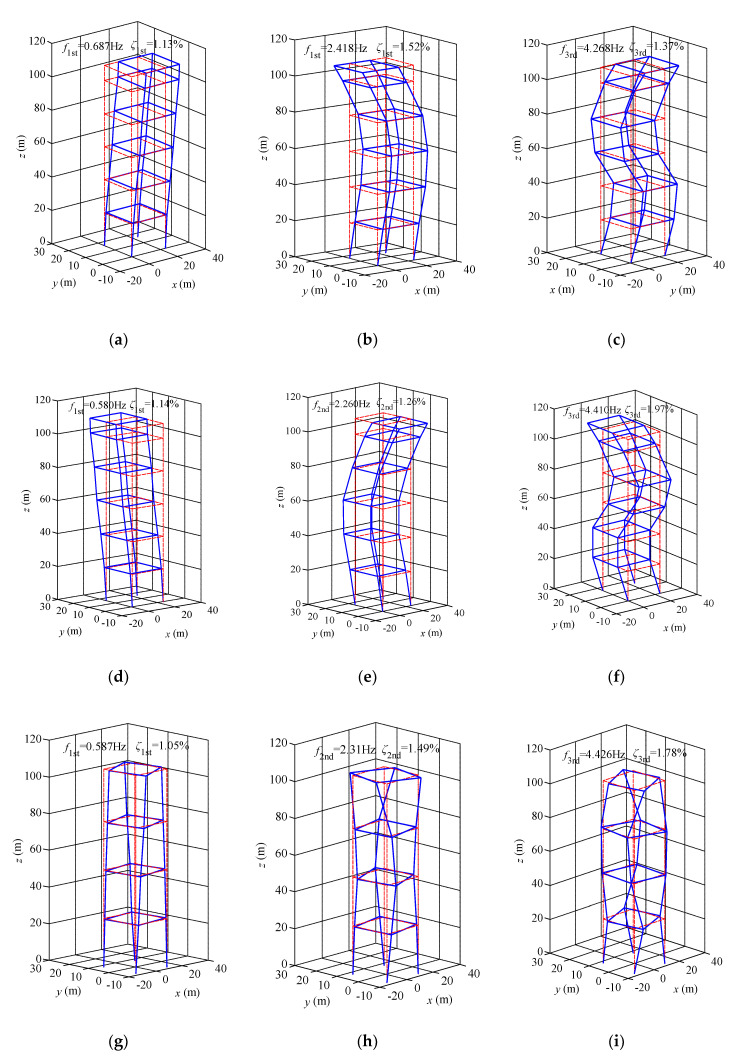
The first three modal parameters under ambient excitations identified by ERA-NExT. (**a**) 1st mode along *x*-axis; (**b**) 2nd mode along *x*-axis; (**c**) 3rd mode along *x*-axis; (**d**) 1st mode along *y*-axis; (**e**) 2nd mode along *y*-axis; (**f**) 3rd mode along *y*-axis; (**g**) 1st mode along torsional direction; (**h**) 2nd mode along torsional direction; (**i**) 3rd mode along torsional direction.

Using ERA-NExT and SSI methods, the translational accelerations of 6th, 12th, 18th, 24th, 30th and 32nd floors and torsional angular accelerations of 8th, 16th, 24th and 32nd floors under ambient excitations are analyzed, and the measured first three modal parameters along the *x*-axis, *y*-axis and torsional direction (T) are obtained. Figure 15 shows that the first three modal parameters of the measured high-rise building under ambient excitations obtained by ERA-NExT method. Table 4 shows the first three modal parameters of the measured high-rise building under ambient excitations identified by ERA-NExT and SSI. In this paper, ambient excitation refers to the high-rise buildings are excited by environmental factors such as normal wind, noise and vibration before typhoon landing, at this stage, the wind-induced response can be approximately used to calculate the modal parameters of high-rise buildings under static wind.

## 5. Amplitude-Dependent Modal Frequency

### 5.1. Sample Selection

When studying the characteristics of modal parameters under typhoon excitation, the wind direction of the 10-min sample changes constantly in the process of typhoon landing, thus the included angle between wind direction and vibration direction of *x* and *y* axes should be strictly controlled to avoid excessive deviation. The wind direction under typhoon Sarika in high wind speed and the large amplitude of dynamic response are prone to the *x*-axis. Therefore, this study regards the wind direction close to the *x*-axis as the along-wind vibration, and the wind direction close to the *y*-axis as the cross-wind vibration. The absolute values of the included angles are strictly controlled from 0° to 10°. It can be approximated that the along-wind load of typhoon mainly affects the *x*-axis vibration response, while the cross-wind load mainly affects the *y*-axis vibration response. Based on the measured dynamic response during Typhoon Sarika, the modal frequencies and damping ratios of translational and torsional directions of high-rise buildings under Typhoon Sarika are identified by ERA-NExT, SSI and RDT (Jeary, et al., 1992 [7]) methods with an interval of 10-min, including the *x*-axis, *y*-axis and torsional dynamic response with different landing stages of Typhoon Sarika.

### 5.2. Modal Frequencies under Different Acceleration Amplitudes

**Figure 16:**
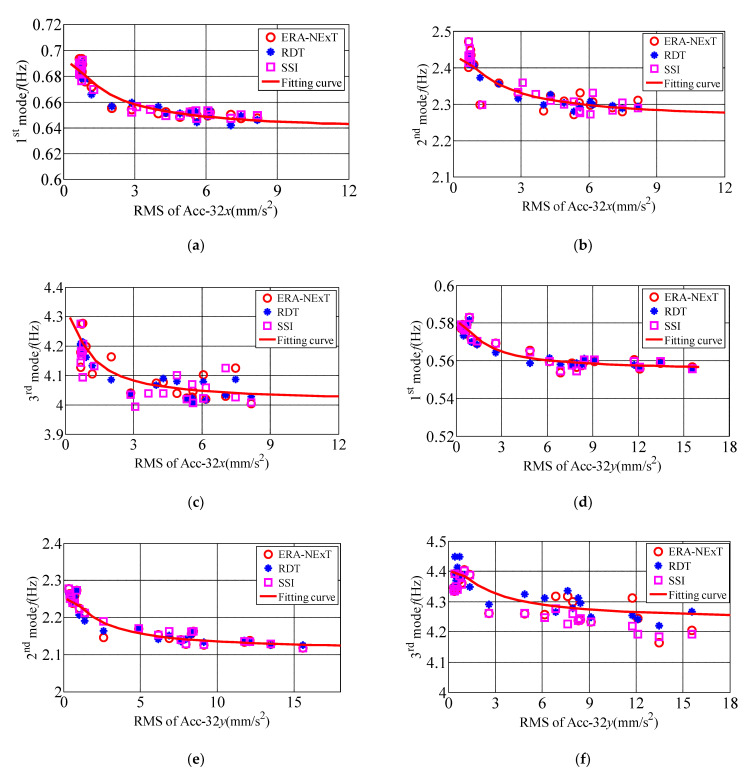

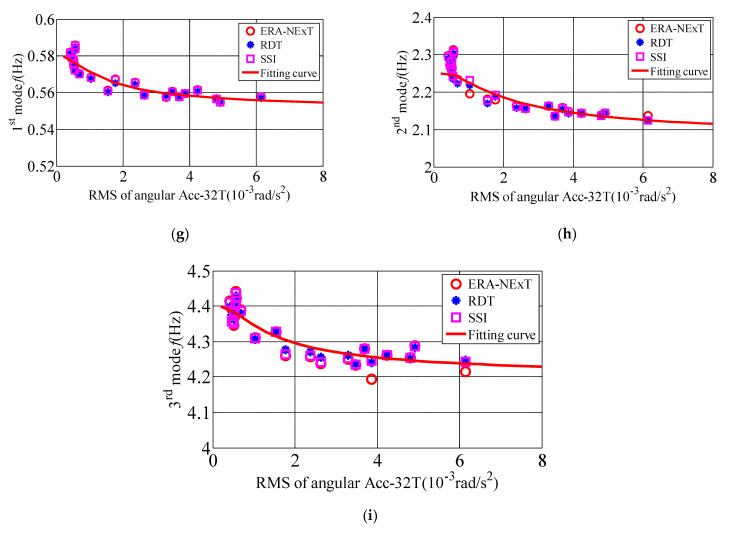
The relationship between translational and torsional modal frequency and RMS of Acc atop the building. (**a**) 1st modal frequency along *x*-axis; (**b**) 2nd modal frequency along *x*-axis; (**c**) 3rd modal frequency along *x*-axis; (**d**) 1st modal frequency along *y*-axis; (**e**) 2nd modal frequency along *y*-axis; (**f**) 3rd modal frequency along *y*-axis; (**g**) 1st modal frequency along torsional direction; (**h**) 2nd modal frequency along torsional direction; (**i**) 3rd modal frequency along torsional direction.

The natural period is the inherent basic dynamic characteristic of high-rise buildings. Figure 16 shows 10-min variations of the amplitude-dependent modal frequency along translational and torsional directions identified by ERA-NExT, SSI, RDT methods. It proves that the recognition results identified by the three methods vary little and follows a similar rule. The measured ranges of modal frequencies at the first three modes are (0.64 0.70) Hz, (2.25 2.50) Hz, (3.95 4.30) Hz along *x*-axis, (0.55 0.59) Hz, (2.10 2.30) Hz, (4.10 4.50) Hz along *y*-axis, and (0.55 0.59) Hz, (2.10 2.35) Hz, (4.15 4.50) Hz along torsional direction in sequence. The first three modal frequencies are estimated to be 0.69 Hz, 2.42 Hz and 4.28 Hz along *x*-axis, 0.58 Hz, 2.28 Hz and 4.40 Hz along *y*-axis, and 0.59 Hz, 2.31 Hz and 4.43 Hz along torsional direction accordingly in the static wind condition. The measured modal frequencies of translational and torsional directions of high-rise buildings under typhoon excitation are lower than those under static wind condition. This phenomenon shows that the measured modal frequencies along *x*, *y* axes and torsional directions are closely related to the acceleration amplitude of the measured high-rise building. When the RMS of translational acceleration is less than 5 mm/s^2^ at the top floor, the modal frequencies of the measured high-rise building along *x* and *y* axes decrease greatly. When the RMS of translational acceleration is greater than 5 mm/s^2^, the reduction rate of frequency decreases. When the RMS of torsional angular acceleration is less than 2 × 10^−3^ rad/s^2^ at the top floor, the torsional modal frequencies of the measured high-rise building decrease sharply. When the RMS of torsional angular acceleration is greater than 2 × 10^−3^ rad/s^2^, the reduction rate of the torsional modal frequency decreases after reaching a critical amplitude. The reductions of the modal frequencies are 7%, 9%, and 7% in sequence along *x*-axis, 7%, 9%, 6% along *y*-axis, and 7%, 9% and 6% in sequence along torsional direction when the translational and torsional acceleration amplitude reaches a maximum value.

### 5.3. Comparison of Mode Shape of the Measured High-Rise Building under Different Wind Speeds

**Figure 17:**
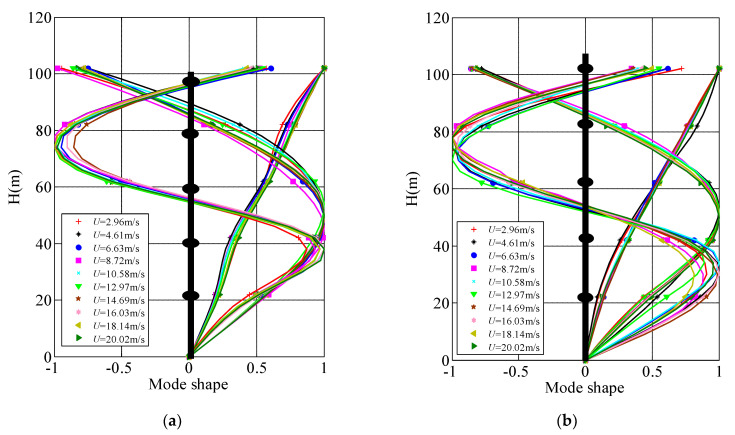
The first three mode shapes along *x*-and *y*-axes under different wind speeds. (**a**) along *x*-axis; (**b**) along *y*-axis.

The translational vibration along *x* and *y* axes is greater than torsional vibration under strong wind, this paper takes modal shapes along *x* and *y* axes as examples for analysis. Taking 10 min as the basic time interval, the first three mode shapes along *x* and *y* axes are analyzed, and the specific mode shape vectors are shown in Figure 17. In order to more accurately identify the similarity of the first three order vibration mode vectors under different wind speeds by ERA-NExT method, Modal Assurance Criterion (MAC) method is used to judge the similarity of mode shape vectors. For example, mode shape vectors obtained from two wind speed samples are {*ϕ*_ai_} and {*ϕ*_ei_} respectively, and the similarity can be determined by the following formula,

(2) $$\mathrm{MAC}(\left\{\phi_{\mathrm{ai}}\right\},\left\{\phi_{\mathrm{ei}}\right\})=\frac{{\left({\left\{\phi_{\mathrm{ai}}\right\}}^{T}\left\{\phi_{\mathrm{ei}}\right\}\right)}^{2}}{\left({\left\{\phi_{\mathrm{ai}}\right\}}^{T}\left\{\phi_{\mathrm{ai}}\right\}\right)\left({\left\{\phi_{\mathrm{ei}}\right\}}^{T}\left\{\phi_{\mathrm{ei}}\right\}\right)}$$

**Figure 18:**
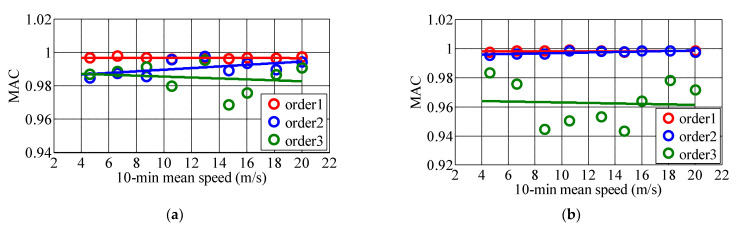
MAC values of measured *U*_10min_ relative to 2.96 m/s in the first three mode shapes with the same order. (**a**) along *x*-axis; (**b**) along *y*-axis.

**Figure 19:**
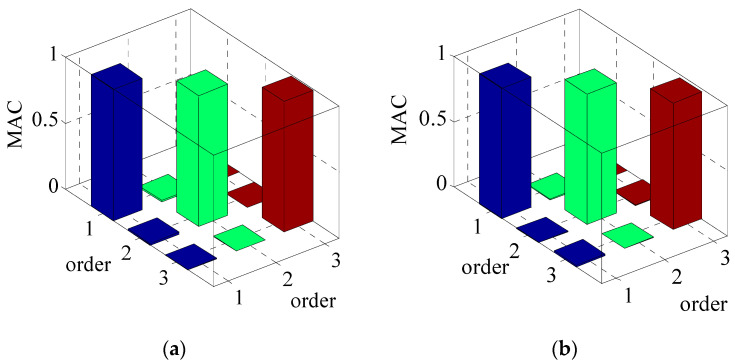
MAC histograms of the first three mode shapes under 10 min wind speeds of 2.96 m/s and 20.02 m/s. (**a**) along *x*-axis; (**b**) along *y*-axis.

The value range of MAC is between [0, 1], if MAC is close to 1, it is considered that there is a certain similarity between the vibration mode shape vectors identified by different wind speed samples; If MAC = 0, it is considered that there is no similarity between the two vectors. The MAC method is adopted to judge the similarity degree of mode shapes under static wind and high wind speed. MAC values of measured *U*_10min_ relative to 2.96 m/s in the first three mode shapes with the same order are shown in Table 5 and Figure 18. Figure 19 presents the MAC histograms of the first three mode shapes under 10 min wind speeds of 2.96 m/s and 20.02 m/s.

Figure 18 reveals that the variations of mode shape vectors at the key points are small, and the mode shapes are basically the same. The maximum deviations of the first three mode shape vector along *x* and *y* axes are all appear on the 24th floor. The maximum deviations of mode shape vectors at the first order along *x* and *y* axes are 0.10 and 0.08, respectively, and those at the second order are 0.25 and 0.15, and those at the third order are 0.18 and 0.25 in sequence. It can be concluded that the deviation of the mode shape vector at the higher orders identified under different wind speeds is more significant than that at the lower orders. Figure 19 shows that the MAC values of the same modal order approach to 1 under different wind speeds. The MAC values of the first three order mode vectors at the same order under relative static wind conditions of different wind speeds are between 0.94 and 1. This discloses that wind speed exerts little influence on the first three mode shape vectors, and the specific identification results are shown in Table 5. The MAC values of vibration mode vector at higher orders fluctuate more considerable than those at lower orders as mode orders increase, which reveals that the variation of wind speed exerts greater influence on mode shape vectors in higher order. 

In sum, the wind speed exerts great influence on mode shape vectors in higher order, while in general the MAC values of the first three mode shape vectors at the same order under relative static wind conditions of different wind speeds are between 0.94 and 1, and the wind speed has little influence on the first three mode shape vectors. 

## 6. Conclusions

Acceleration responses along translational and torsional directions are monitored synchronously during the passage of Typhoon Sarika in Haikou. A large number of samples covering different wind directions are obtained. The first three modal parameters of the measured high-rise building along translational and torsional directions are investigated. The variation characteristics of amplitude-dependent modal frequency of the high-rise building along translational and torsional directions are proposed to make up for the deficiency of the measured pure torsional vibration response of high-rise buildings. The main conclusions are as follows:

(1) The root-mean-square of accelerations and peak values along both the longitudinal, lateral and torsional directions vary in an increasing power function with the increasing wind sped. The peak value of the self-power spectrum corresponding to the mode frequency of the *y*-axis (weak-axis along cross-wind direction) is obviously larger than that of the *x*-axis (strong-axis along-wind direction) self-power spectrum, which indicates that the magnitude of the acceleration is related to the energy of the vibration direction, and the dynamic response of cross-wind direction under typhoon action can be greater than that along-wind direction under certain conditions. The peak values of Auto-power spectral density (APSDF) corresponding to the first three modal frequency of translational and torsional directions increase significantly with the wind speed increases during the passage of typhoons, especially the peak values of APSDF at the first modal frequency. This indicates that the first mode is the dominant mode under typhoon action. The frequencies corresponding to the peak values of APSDF under the ambient excitations is obviously bigger than that under the action of strong wind, this phenomenon shows the translational and torsional amplitude-dependent modal frequency of first three orders decrease significantly with the wind speed increases.
(2) The measured modal frequencies of longitudinal, lateral and torsional directions are closely related to the acceleration amplitude of the measured high-rise building. When the RMS of translational accelerations are less than 5 mm/s^2^ at the top floor, the modal frequencies of *x* and *y* axes of the measured high-rise building decrease relatively large. When the RMS of translational accelerations are greater than 5 mm/s^2^, the frequency reduction rate reduces after reaching a critical amplitude. When the RMS of torsional angular accelerations are less than 2 × 10^−3^ rad/s^2^ at the top floor, the torsional modal frequencies of the measured high-rise building decrease relatively large. When the RMS of torsional angular accelerations are greater than 2 × 10^−3^ rad/s^2^, the torsional modal frequency reduction rate reduces after reaching a critical amplitude. The first three modal frequencies reduces 7%, 9%, 7% in sequence along *x*-axis, and 7%, 9%, 6% in sequence along *y*-axis, and 7%, 9%, 6% in sequence along torsional direction when the translational and torsional acceleration amplitude reaches a maximum value.

## Figures and Tables

**Table 1:** Specifications of the anemometer and acceleration sensors.

Monitoring Equipment	Sensor Range	Resolution	Operating Temperature Range	Size	Weight	Equipment Photos
RM.Young 05103V	0~100 m/s 0~360°	±0.3 m/s ±3°	−50~50 °C	High 37 cm Long 55 cm Propeller diameter 180 mm	1.0 kg	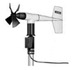
991B translational accelerometer	0~±20 m/s^2^	5 × 10^−6^ m/s^2^	−10~+50 °C	72.5 mm × 72.5 mm × 88 mm	0.6 kg	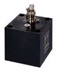
RA013 rotational accelerometer	0~±5 rad/s^2^	2 × 10^−4^ rad/s^2^	−20~+70 °C	330 mm × 130 mm × 105 mm	6 kg	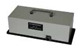

**Table 2:** The fitting results of translational parameters of c_1_ and c_2_.

Acceleration	Direction	Parameters	6th Floor	12th Floor	18th Floor	24th Floor	30th Floor	32nd Floor
RMS of Acc	*x*	c_1_	0.006	0.012	0.011	0.011	0.016	0.019
		c_2_	1.849	1.813	1.962	2.038	1.996	2.008
	*y*	c_1_	0.012	0.004	0.003	0.003	0.004	0.008
		c_2_	1.706	2.335	2.587	2.656	2.647	2.468
Peak acceleration	*x*	c_1_	0.176	0.306	0.531	0.297	0.159	0.463
		c_2_	1.258	1.249	1.147	1.422	1.742	1.474
	*y*	c_1_	0.262	0.070	0.067	0.107	0.115	0.715
		c_2_	1.216	1.869	2.029	1.968	2.038	1.508

**Table 3:** The fitting results of torsional parameters of c_1_ and c_2_.

	Parameters	c_1_	c_2_	c_3_
RMS of angular-Acc	8th floor	8.11 × 10^−6^	−1.45 × 10^−4^	0.0011
	16th floor	1.18 × 10^−5^	−1.76 × 10^−4^	0.0012
	24th floor	2.44 × 10^−5^	−3.95 × 10^−4^	0.0028
	32nd floor	1.85 × 10^−5^	−1.50 × 10^−4^	0.0006
Peak angular acceleration	8th floor	4.16 × 10^−5^	−0.0009	0.0086
	16th floor	5.84 × 10^−5^	−0.0012	0.0108
	24th floor	1.69 × 10^−5^	−0.0040	0.0340
	32nd floor	1.79 × 10^−5^	−0.0012	−0.0089

**Table 4:** The first three modal parameters of the measured high-rise building under ambient excitation identified by ERA-NExT and SSI.

Mode Number	Vibration Direction	NExT-ERA		SSI	
		$f_{}\;(\mathrm{Hz})$	$\zeta_{}\;(\%)$	$f_{}\;(\mathrm{Hz})$	$\zeta_{}\;(\%)$
1	The translational 1st mode along *y*-axis	0.580 Hz	1.14%	0.581 Hz	1.12%
2	The torsional 1st mode	0.587 Hz	1.05%	0.587 Hz	0.96%
3	The translational 1st mode along *x*-axis	0.687 Hz	1.13%	0.687 Hz	1.06%
4	The translational 2nd mode along *y*-axis	2.260 Hz	1.26%	2.260 Hz	1.38%
5	The torsional 2nd mode	2.310 Hz	1.49%	2.310 Hz	1.27%
6	The translational 2nd mode along *x*-axis	2.418 Hz	1.52%	2.420 Hz	1.48%
7	The translational 3rd mode along *x*-axis	4.268 Hz	1.37%	4.262 Hz	1.53%
8	The translational 3rd mode along *y*-axis	4.410 Hz	1.97%	4.402 Hz	1.54%
9	The torsional 3rd mode	4.426 Hz	1.78%	4.430 Hz	1.15%

**Table 5:** MAC values of measured *U*_10min_ relative to 2.96 m/s in the first three mode shapes with the same order.

Along *x*-Axis (*U*_10min_ Relative to 2.96 m/s)						Along *y*-Axis (*U*_10min_ Relative to 2.96 m/s)					
Order 1		Order 2		Order 3		Order 1		Order 2		Order 3	
*U*_10min_	MAC	*U*_10min_	MAC	*U*_10min_	MAC	*U*_10min_	MAC	*U*_10min_	MAC	*U*_10min_	MAC
4.61 m/s	0.997	4.61 m/s	0.985	4.61 m/s	0.987	4.61 m/s	0.998	4.61 m/s	0.996	4.61 m/s	0.983
6.63 m/s	0.998	6.63 m/s	0.987	6.63 m/s	0.989	6.63 m/s	0.999	6.63 m/s	0.996	6.63 m/s	0.976
8.72 m/s	0.997	8.72 m/s	0.985	8.72 m/s	0.991	8.72 m/s	0.998	8.72 m/s	0.996	8.72 m/s	0.944
10.58 m/s	0.996	10.58 m/s	0.995	10.58 m/s	0.980	10.58 m/s	0.999	10.58 m/s	0.999	10.58 m/s	0.950
12.97 m/s	0.996	12.97 m/s	0.998	12.97 m/s	0.995	12.97 m/s	0.998	12.97 m/s	0.998	12.97 m/s	0.953
14.69 m/s	0.996	14.69 m/s	0.989	14.69 m/s	0.969	14.69 m/s	0.998	14.69 m/s	0.998	14.69 m/s	0.943
16.03 m/s	0.997	16.03 m/s	0.993	16.03 m/s	0.976	16.03 m/s	0.999	16.03 m/s	0.999	16.03 m/s	0.964
18.14 m/s	0.996	18.14 m/s	0.990	18.14 m/s	0.987	18.14 m/s	0.998	18.14 m/s	0.999	18.14 m/s	0.978
20.02 m/s	0.997	20.02 m/s	0.994	20.02 m/s	0.991	20.02 m/s	0.998	20.02 m/s	0.998	20.02 m/s	0.972

## Data Availability

Not applicable.
